# Integrated Care: Better and Cheaper: Schrijvers, G. (2017). Amsterdam: Reed Business Information

**DOI:** 10.5334/ijic.4730

**Published:** 2019-08-22

**Authors:** Liliane Rocha

**Affiliations:** 1 CUMMINGS Graduate Institute for Behavioral Health Studies, US

**Keywords:** Triple Aim, Integrated care, capuccino model, population health

Integrated care: better and cheaper, Schrijvers (2017) is an excellent book for those interested in learning about the foundations of integrated care and how it addresses many challenges in the healthcare industry. While the author suggests that the book is aimed at professionals, managers, and health care policy-makers, I would propose that it can also be an asset in an introductory graduate level course on integrated care. Throughout the book, the reader will be stimulated by Schrijvers’s arguments on how integrated care contributes to the actualization of the Triple Aim—i.e., enhancing population health and improving quality of care (better), while lowering cost per capita (cheaper). Discussing hundreds of articles as evidence that integrated care is, in fact, better and cheaper than traditional non-integrated care, Schrijvers presents the evidence along six dimensions: type of integrated care, patients as partners, quality of integrated care, paying integrated care digitization and integrated e-health, and leadership, innovation and research of integrated care (integrated care policies).

The book is divided in parts, each representing one of the dimensions of integrated care cited above. In the introductory chapters, the author presents basic concepts and technical terms used throughout the book, and familiarizes the reader with the basic question of the book: “*Is it possible to improve population health, increase quality of care for the individual and lower per capita costs of care using person-centered integrated care*?” In each subsequent chapter, Schrijvers describes evidence from research and from his own experience in healthcare in the Netherlands, such that the reader can appreciate how integrated care satisfies the aspirations of the Triple Aim. In part 2, Types of Integrated Care, Schrijvers discusses the benefits of horizontal integration, vertical integration, case management, pharmaceutical care and integration with social services. The evidence points to lower cost per capita associated with each of these different areas of integrated care, as well as improvement in population health and better quality of care for the individual.

In part 3, the partnership between patients and providers within integrated care is examined. The author discusses how changes in behavior associated with developments in society, such as improvement in hygiene, have a greater impact on public health than the development of medical science. He argues that the individual is the greatest change agent of his own health, and he postulates that integrated care can enhance the success of self-management and shared decision-making. In part 4, Schrijvers presents different perspectives on quality of integrated care; that of the professional and that of the patient. He proposes variables associated with each perspective, and ways that organizations can measure advances associated with lowering costs and improvements in patient experience. In part 5, financing integrated care is discussed. Schrijvers discusses how behavioral economics concepts can explain decision-making processes in the set-up of healthcare programs, and how to use these concepts and strategies for service improvement and lowering costs. He presents the “*cappuccino model*”, which is essentially a combination of three layers (population-based payment, a low fee-for-service, and a low fee for innovation or pay for performance). In addition, he discusses the use of integrated personal budget funding system.

In part 6, advances in digitization and e-health such as changes to the electronic health record (EHR) and practical issues in e-health are examined. Schrijvers proposes that data derived from EHR can help in the design of population health programs. In part 7, the role of leadership in supporting integrated care is explored along with research principles in integrated care. In the conclusion, a summary table is presented with the findings discussed in each chapter as they are applied to the main question of the book, giving the reader a place for quick referencing concepts discussed in the chapters.

While these topics are presented separately, the author emphasizes how these concepts are interconnected and directs the reader to related resources introduced in the book. The explicit focus of the book was the Triple Aim, however, the reader can also infer from the book’s examples how integrated care benefits the fourth aim, care of the provider, as well. Those who are beginning their journey in integrated care will find in this book a well-organized presentation of its value proposition. Those who are more seasoned in integrated care will find inspiration and assurances that integrated care is the way to bring care that is better for the individual and cheaper for payers. However, if one is looking for a book that will suggest specific ways to improve integrated care such that it will be cheaper, as the title might suggest, this is not the book for you. It does not discuss case studies on implementation, from which one can derive strategies to be applied in the settings where they work. Instead, it supports the idea that if care is planned and delivered in an integrated way, it will result in better care for the patient, it will address issues related to population health, and it can lower costs. Rather than proposing a new administrative or organizational model, Schrijvers sticks to the facts to make the case that integrated care is a viable response to the goals established with the Triple Aim.

